# Perceptions and attitudes of kidney supportive care among elderly patients with advanced chronic kidney disease and dialysis healthcare professionals in China: a qualitative study

**DOI:** 10.1186/s12882-023-03372-2

**Published:** 2023-10-26

**Authors:** Xue Li, Jin Kai Luo

**Affiliations:** Beijing Friendship Hospital, Nursing Department, Beijing Friendship Hospital, Capital Medical University, Capital Medical University, NO. 95 Yong-An Road, Xi-Cheng District, Beijing, 100050 PR China

**Keywords:** Kidney supportive care, Palliative care, ESRD, Elderly patients, Qualitative study

## Abstract

**Purpose:**

Kidney supportive care (KSC) represents a novel approach wherein a multidisciplinary team of nephrology experts offers active symptom management, advance care planning, shared decision-making, conservative treatment, and end-of-life care. This study is aimed at providing comprehensive insights and evaluations regarding the understanding and perspectives of Chinese healthcare professionals, including nephrologists and dialysis nurses, as well as elderly individuals aged 60 and above who are afflicted with chronic kidney disease. The primary goal is to furnish substantial information support for a better comprehension of KSC within the Chinese context, with the ultimate aim of facilitating its effective implementation in this region.

**Methods:**

Employing a phenomenological framework, this qualitative research generated data using semi-structured interviews with 18 elderly patients diagnosed with advanced chronic kidney disease and 10 healthcare professionals across two Class III Grade A medical institutions. The Colaizzi’s analytical method was utilized for coding and analyzing the interview data.

**Results:**

Distinct interviews were executed with patients and healthcare professionals to delineate specific themes for each group. For elderly dialysis patients, the following themes were formed: (1) Lack of understanding of KSC; (2) Concerns of how KSC is perceived; and (3) Perceived benefits of KSC. Within the healthcare professionals cohort, two salient themes emerged: (1) Prospective clinical merits of KSC ; and (2) Mltiple difficulties encountered.

**Conclusions:**

This study goes some way in furnishing a holistic understanding of perceptions surrounding KSC among elderly patients undergoing dialysis and healthcare professionals in China. The overarching Chinese cultural ethos poses substantial challenges to the widespread assimilation of KSC. While healthcare professionals advocate for KSC, there remains a limit in the patients’ comprehension of this therapeutic paradigm. There is a compelling imperative to cultivate this strategy, grounded in the prevailing medical landscape.

## Introduction

The prevalence rate of chronic kidney disease (CKD) in the elderly population(Age ≥ 60) in China is currently 30–50% [1]. Consequently, the aging population with End-Stage Renal Disease (ESRD) contends with a heightened individual disease burden.Protracted dialysis treatment often subjects patients to a myriad of symptoms, including pain, fatigue, nausea, anorexia, and insomnia. This, in turn, compromises their nutritional and physical health, inducing concomitant psychological maladies like anxiety and depression [2, 3]. It is evident that elderly patients with ESRD enduring prolonged dialysis manifest varying degrees of cognitive impairment, with resultant deterioration in their physical function and nutritional health [4]. Furthermore, the exigencies of sustained treatment for ESRD result in exorbitant medical costs, exerting substantial financial strain on patients and their familial support structures [5]. Elderly ESRD patients, particularly those who can no longer endure dialysis, experiene pronounced declination in their living standards and quality of life. Intriguingly, for patients exceeding 80 years, the survival advantages of dialysis are often eclipsed by the merits of robust supportive care and holistic symptom management [6]. Empirical studies advocate that such proactive supportive care, particularly for aging patients or those disinterested in continuing prolonged dialysis, has affirmative implications for enhancing ESRD patients’ quality of life [7].

Recent academic explorations exhibit a burgeoning interest in KSC, underscored by the impetus to ameliorate disease-associated distress and augment the quality of life during terminal stages [8]. With escalating awareness in patients’ prognosis, the emergence of palliative cognizance, and the advocacy for shared decision-making, KSC unveils a revolutionary therapeutic alternative for ESRD patients. KSC is specifically tailored for patients battling advanced Renal conditions or those undergoing any dialysis modality, inclusive of those with acute Renal afflictions or those who have opted for kidney replacement modalities [9].This therapeutic modality marries the acumen of Renal and palliative care specialists and is orchestrated by a collaborative healthcare professionals comprising nephrologists, nurses, and palliative care clinicians. The chief aim is to ameliorate symptoms and improve the quality of life for terminal-stage Renal patients [10].

KSC is an update and development of palliative care for patients with kidney disease. KSC includes the decision to withdraw from dialysis based on shared decision-making with the patient and family, followed by the provision of active clinical symptom management, detailed prognosis communication, advanced care planning and end-of-life care palliative planning, and comprehensive psychosocial and spiritual support [6, 11]. KSC is not merely a patient-centric paradigm for symptom management or a straightforward palliative care avenue. Instead, it amplifies the scope of palliative care for ESRD patients by introducing an early, interdisciplinary, and encompassing care module [12].

Presently, the adoption of KSC is predominantly observed the United Kingdom, Canada, the United States, Australia, and New Zealand [13]. However, its integration within China remains in its infancy [13]. The model has yet to be seamlessly incorporated into the care framework for ESRD patients, the impediments to its evolution in China include a conspicuous absence of robust policy backing, a deficiency in specialized training initiatives, and fiscal safeguard mechanisms [14]. Thus, while the concept of palliative care and KSC has been tentatively explored in regions like Hong Kong and Taiwan, the mainland offers limited empirical studies on the subject [15].

In light of this backdrop, there is an undeniable imperative to critically evaluate perceptions and attitudes concerning the potential and viability of this innovative approach. Before championing its full endorsement within the Chinese medical community, encompassing patients, and healthcare professionals, a thorough understanding is required. Therefore, this study employs a qualitative interview approach with the aim of providing comprehensive insights and evaluations regarding the perspectives of Chinese healthcare professionals and patients on KSC. The primary objective is to enhance the understanding of KSC within the context of China, ultimately promoting its effective implementation in clinical practice.

## Methods

### Background context

This investigation employed a qualitative, exploratory, descriptive approach, utilizing semi-structured interviews anchored in phenomenological research principles, guided by Colaizzi’s seven-step analytical framework [16]. This method delves into experiences, elucidating the conceptual perception of a patient’s ailment and by furnishing a holistic representation of an experiential phenomenon, uncovers its fundamental architecture. Beyond mere description, this method interprets the underlying essence of the phenomenon [17]. This study rigorously adheres to established standards of qualitative research [18]. The focus of the interviews is to provide comprehensive insights and evaluations regarding the perspectives of Chinese healthcare professionals and patients on KSC. The primary objective is to enhance the understanding of KSC within the context of China and ultimately promote its effective implementation in clinical practice.The reporting of this research adheres to COREQ standards [19].

### Setting and participants

Interview sessions spanned from August 2022 to February 2023. Participants were recruited from nephrology departments and a hemodialysis center across two tertiary, Class a hospitals situated in Zhengzhou and Beijing, China. Interviewee participation was both voluntary and predicated on objective sampling. Demographic details collected from healthcare professionals included: age, gender, occupation type, educational attainment, and tenure of professional experience and patients’ demographics were age, gender, education background, dialysis type, dialysis duration, monthly household income and marital status.

Ultimately, 10 healthcare professionals, comprising 3 physicians and 7 nurses, were recruited. Alongside, 18 elderly patient with ESRD receiving dialysis were interviewed, culminating in a cohort of 28 participants. Healthcare professionals eligibility criteria included: (1) Possession of a bachelor’s degree or higher; (2) A minimum of 5 years of nephrology-centric work experience; (3) Availability for interview participation. (4)Informed consent and voluntary participation in the study.Exclusion Criteria: Absence from duty due to illness or personal leave.Patient inclusion criteria encompassed: (1) Diagnosis in alignment with K/DOQI guidelines for ESRD [20]; (2) Age ≥ 60;(3) Effective communication capacity; (4) Absence of cognitive impairment; (5) Providing informed consent and expressing volition to partake. Exclusion criteria entailed: (1) Severe complications or conditions hindering participation; (2) Keep in pronounced communication barriers.

### Data Collection

Demographic details collected from healthcare professionals included: age, gender, occupation type, educational attainment, and tenure of professional experience and patients’ demographics were age, gender, education background, dialysis type, dialysis duration, monthly household income and marital status. The study’s interview guide was developed post thorough examination of pertinent academic literature and research studies [21, 22]. A qualitative research-oriented nursing team, inclusive of one professor and two scholars, vetted the questions. To refine the interview schema, preliminary sessions were conducted with three healthcare professionals and three ESRD patients. The modified interview template was then deployed comprehensively. The final detailed interview scheme can be found in Table 1.

**Table 1:** Interview Questions

Interview participants	Interview Questions
For Patients	• When you hear the term KSC^a^, what do you think this means? • If KSC is implemented in the clinic for elderly patients with kidney disease, what effect do you think it will have ? • Under what circumstances do you think you would choose KSC? • What do you think is your family’s attitude towards your acceptance of KSC? • Do you have any concerns or concerns about using KSC?
For Healthcare professionals (nephrologists and dialysis nurses)	• Combined with the current clinical practice in China, what is your opinion and attitude towards the implementation of KSC for end-stage elderly patients with kidney disease? • What obstacles do you think would exist if KSC were implemented for elderly patients with advanced kidney disease in China? • What factors do you think can promote the implementation of KSC in elderly patients with kidney disease in China?

^a^KSC – Kidney Supportive Care

Two qualified researchers administered the interviews, using semi-structured guidelines, and capturing audio recordings within the confines of a hospital consultation room [23]. Before initiating each session, participants were oriented thoroughly to the core tenets and objectives of KSC. Interviews were carried out in a gradual manner, commencing with broad topics and progressed gradually to more specific issues.Interviews unfurled in a gradual manner, commencing with broad queries and progressing fluidly. A congenial environment was maintained throughout, with researchers utilized techniques such as probing, active listening, and repetition to stimulate candid discourse. Individual sessions spanned approximately 20–50 min. Not only were verbal expressions documented, but also non-verbal cues like tone modulation, facial dynamics, ocular shifts, and body language. Data saturation was deemed achieved when no new code was extracted, signaling the interview’s conclusion.

### Data analysis

Within the scope of our investigation, two distinct demographic groups were solicited for inputs, necessitating differential interview guides tailored for each cohort. During the analytical phase, the testimonies from each group were meticulously dissected, adopting the structured Colaizzi’s seven-step analysis methodology for data interpretation [24]. Each audio recording of interviews was attentively revisited by the lead investigator and transcribed verbatim. The analytical process guided by Colaizzi’s methodology was as follows [25]: (1) Immersing oneself by repeatedly reading participant narratives to grasp the underlying sentiments; (2) Highlighting and enumerating pivotal statements; (3) Translating these pivotal statements into academic lexicon; (4) Aggregating and categorizing similar emergent concepts or themes; (5) Structuring sub-themes from the collated data; (6) Hierarchically organizing these sub-themes into overarching thematic umbrellas; (7) In a bid for analytic veracity, these synthesized interpretations were revisited with the participants. However, there was no need for reiteration or second interviews. The collective expertise of the research team was harnessed to bolster the credibility of the findings and facilitate deeper insights. NVivo11.4.2 software was used to assist with data management and analysis.

### Ethics approval and consent to participate

This study was conducted in accordance with the principles contained in the Guidelines for Good Clinical Practice in the Declaration of Helsinki. This study was approved by the Ethics Committee of Beijing Friendship Hospital Affiliated to Capital Medical University. All subjects agreed to participate in the study after being informed orally and in writing of the objectives and procedures of the study. Informed consent was obtained from all participants.

## Results

### Study sample

From an initial cohort of 25 approached patients, 20 were successfully recruited. Subsequent attrition occurred with one participant voluntarily exiting prior to data capture, while another had to withdraw due to transferring to another dialysis facility.This led to a final total of 18 patient subjects. Of these, thirteen were associated with medical institutions in Zhengzhou, and the remaining five were from Beijing-based hospitals. Of the 15 healthcare professionals initially approached, 13 consented to participate. However, occupational commitments precluded the participation of three, resulting in 10 healthcare professionals participating. Within this group, six hailed from Zhengzhou hospitals, while the other four were affiliated with Beijing hospitals.

In the patient sample, ages ranged from 61 to 87 years. The cohort comprised 12 males and 6 females. While the majority (15) were undergoing hemodialysis, the remaining three were on peritoneal dialysis, and their dialysis experience varied from 1 to 11 years (refer to Table 2). Among 10 healthcare professionals, the composition was three physicians and seven nephrological nurses. A predominant female representation was observed (7 out of 10). All healthcare professionals had at least a bachelor’s degree, and professional tenures varied from 8 to 30 years (refer to Table 3).The interview duration for both groups of participants ranged from 20 to 50 min. The average interview duration for patients was 36.52 min, while healthcare professionals had an average interview duration of 34.39 min.

**Table 2:** Characteristics of patient participants

NO	Gender	Age (years)	Type of dialysis	Dialysis Duration (years)	Educational level	Economic level Income per month (RMB)	marital status	Number of complications	Residence	Interview duration in minutes
Patient 1	Female	65	Peritoneal dialysis	3	Primary school	1000 ~ 3000	Married	2	Villages and towns area	40.10
Patient 2	Male	78	hemodialysis	7	High school	3000 ~ 5000	Married	3	Urban area	35.33
Patient 3	Male	80	hemodialysis	10	Middle school	3000 ~ 5000	Married	3	Urban area	39.12
Patient 4	Female	63	Peritoneal dialysis	1	High school	3000 ~ 5000	Married	0	Urban area	40.05
Patient 5	Male	84	hemodialysis	9	Primary school	1000 ~ 3000	Married,widowed	2	Villages and towns area	29.55
Patient 6	Male	65	hemodialysis	5	Junior high school	3000 ~ 5000	Married	3	Urban area	38.04
Patient 7	Male	60	hemodialysis	4	Primary school	1000 ~ 3000	Married	2	Rural area	37.29
Patient 8	Female	77	hemodialysis	8	College degree	> 5000	Married	1	Urban area	40.01
Patient 9	Male	79	hemodialysis	9	High school	> 5000	Married	2	Urban area	36.42
Patient 10	Female	65	hemodialysis	3	Junior high school	3000 ~ 5000	Married	1	Urban area	39.59
Patient 11	Male	78	hemodialysis	11	Middle school	3000 ~ 5000	Married	3	Urban area	25.49
Patient 12	Female	69	Peritoneal dialysis	4	Junior high school	1000 ~ 3000	Married,divorced	2	Villages and towns area	38.17
Patient 13	Male	61	hemodialysis	6	College degree	> 5000	Married	3	Urban area	34.39
Patient 14	Male	78	hemodialysis	9	High school	3000 ~ 5000	Married	3	Urban area	40.15
Patient 15	Male	87	hemodialysis	10	College degree	3000 ~ 5000	Married	2	Urban area	40.04
Patient 16	Male	69	hemodialysis	3	College degree	> 5000	Married	1	Urban area	39.18
Patient 17	Female	62	hemodialysis	5	Primary school	1000 ~ 3000	Married,divorced	3	Rural area	34.13
Patient 18	Male	71	hemodialysis	8	High school	3000 ~ 5000	Married	2	Urban area	30.29

**Table 3:** Characteristics of healthcare professionals

NO	Gender	Age (years)	Type of work	Working years	Educational level	Interview duration in minutes
Nurse 1	Female	35	Hemodialysis Nurse	13	Bachelor degree	29.13
Nurse 2	Male	40	Hemodialysis Nurse	20	Postgraduate degree	36.44
Nurse 3	Female	41	Dialysis Nurse	20	Postgraduate degree	40.15
Nurse 4	Female	36	Hemodialysis Nurse	12	Bachelor degree	39.33
Nurse 5	Female	45	Hemodialysis Nurse	22	Postgraduate degree	28.17
Nurse 6	Male	43	Dialysis Nurse	20	Bachelor degree	32.13
Nurse 7	Female	50	Hemodialysis Nurse	25	Bachelor degree	35.26
Doctor 1	Female	38	Dialysis Doctor	10	Doctor’s degree	29.18
Doctor 2	Male	55	Dialysis Doctor	30	Doctor’s degree	35.57
Doctor 3	Female	35	Dialysis Doctor	8	Doctor’s degree	38.49

### Interview results

The respondents were two distinct group: dialysis patients and healthcare professionals. The resulting insights have been systematically cataloged under two broad sections. For dialysis patients, the data included three thematic constructs: “Lack of Perception of KSC” ,“Difficulty in Acceptance” and “Potential Consideration.“ The healthcare professionals’ inputs culminated in two overarching themes:“Prospective clinical merits of KSC”,“Mltiple difficulties encountered” .

## Part I

### Lack of understanding of KSC

#### Limited familiarity with KSC

Discrepancies in medical exposure, educational attainment, and socio-environmental factors underscored some patients’ misconceptions about KSC. One respondent articulated, “*I’m largely uninformed about such therapies and rarely engage with them.*“ (P1) Echoing this sentiment, a senior participant with limited formal education remarked, “Literacy challenges prevent me from grasping the presented medical literature or understanding the conveyed medical advice.“ (P5) A recurring thread was patients’ dependency on physicians for treatment directives. This was evident in the assertion, one patient explained, “Upon hospitalization, we predominantly heed the physician’s counsel, often unaware of alternative therapies like the one you mentioned.” (P1) After a thorough review of KSC’s tenets, several participants voiced their struggle to comprehend its therapeutic implications. An elderly respondent expressed, “The intricacies and potential benefits of this treatment elude me. I often defer to external guidance in such matters.“ (P6) This highlights the paramount influence of patients’ comprehension of KSC on its clinical integration.

#### Low acceptance of KSC

While many patients exhibited an optimistic outlook upon their introduction to KSC, several remained complacent, particularly those content with their present health status and quality of life. One patient, a year into dialysis, remarked, “Having undergone dialysis for a year, I find myself in good health with substantial familial support. I have yet to contemplate these particularities.” (P4) This sentiment was echoed by another, “Currently, I deem my quality of life commendable, coupled with my stable health condition and unwavering family support. I don’t wish to broach this subject with my kin at this juncture.” (P8) Notably, patients in sound health, bolstered by familial backing, tend to allocate minimal thought to therapy alterations or advancements, rendering their acceptance of KSC limited due to an inadequate grasp of its potential benefits.

#### Lack of KSC information support

A significant portion of patients disclosed unfamiliarity with KSC until our interviews. A common notion expressed by participants was the limited avenues available for them to acquaint themselves with novel therapeutic paradigms and concepts in their daily lives, as emphasized by three patients(P2,P5,P6).As P2 elucidated, “I am at a loss regarding avenues to access such content. I seldom operate smartphones and haven’t encountered any televised information pertaining to Renal care.” Especially for those hailing from distant rural locales, economic constraints and environmental factors compound the informational vacuum. A long-time hemodialysis patient from a regional hospital highlighted, “While hospitalized, healthcare professionals seldom introduce us to these novel therapeutic strategies, leaving us clueless about self-educational prospects.” (P7) This was further exemplified by a senior patient who noted, “Given our ineptitude with smartphones, we predominantly rely on our children for queries, who due to their occupational preoccupations seldom engage with us regarding novel Renal therapies.” (P3) This highlights a stark gap in informational infrastructure and unequal medical resource allocation, underscoring the need for targeted professional interventions to foster KSC understanding and assimilation.

### Concerns of how KSC is perceived

#### View of life under traditional culture

Deeply entrenched in traditional Chinese values surrounding life, death, and perseverance, patients, even amidst heightened disease burdens and diminished quality of life, are disinclined to abandon treatment. Their tenacity stems from a fervent desire for longevity. An individual, enduring many years of dialysis, asserted, “I remain unwavering in my commitment to dialysis, nurturing hope for as long as a glimmer exists. I fervently wish to witness the birth of my grandchild and partake in their upbringing.” (P10) Another, having undergone dialysis for a decade and experiencing multiple medical interventions, remarked, “Despite occasional physical torment, my determination to live remains unshaken. I am inclined to persist with treatment regardless of intermittent pain.” (P15) Concurrently, the linchpin of familial support fortifies these patients’ resolve, as aptly voiced by P9, “Even in the throes of pain, our family’s economic stature enables treatment continuity, bolstered by unwavering support. I harbor unwavering faith in medical advancements and remain steadfast.”

#### Lack of autonomy in family relationships

Influenced by enduring traditional family dynamics, certain patients exhibit a conspicuous absence of autonomous decision-making capabilities. This characteristic strongly influences their incentives and outcomes when confronted with pivotal decisions. A patient undergoing dialysis for over a decade elucidated, “My familial support has been unwavering. Decisions regarding my treatment are collaborative familial endeavors, and I’m reticent to modify them without the collective agreement of my family.“ (P11) Another, navigating the tumultuous waters of chronic illness for several years, reflected, “Given the disease’s inherent agonies, I frequently ponder my future. However, crucial decisions are typically contingent upon my wife’s counsel. Given her role as the primary caregiver to both me and our family, her considerations are paramount in my decision-making process.“ (P13, with a somber intonation).

### Perceived benefits of KSC

#### Beneficial for Disease management

Following extended dialysis sessions, the amalgamation of physical discomfort and psychological anguish is palpable amongst most patients. KSC’s emphasis on proactive symptom management and forward-looking interventions resonates deeply with them. One patient, after a 4-year stint with peritoneal dialysis culminating in an infection-induced switch to hemodialysis, lamented, “Bound to these machines, exhaustion pervades my existence. Daily, I grapple with myriad complications. The allure of a serene exit, devoid of further medical interventions, is strong.“ (P12) Another patient, with an academic background and 3 years of dialysis experience, professed, “I aspire for palliative care as my life approaches its twilight. Familiar with advance care planning, I ardently wish to preserve my medical autonomy. In my final moments, I prioritize enhancing the quality of my life over merely extending its duration.” (P16) An individual, upon 10 years ofdialysis with an additional year of uremia and resultant paraplegia, stated, “Traditionally, my family spearheads decision-making. Yet, with my deteriorating quality of life and having endured intense medical procedures, my inclination is towards ceasing treatment and exploring KSC, yearning for a semblance of comfort and solace in my remaining days.“ (P14) This underscores the potential of KSC to alleviate symptom burdens, as patients juxtapose their understanding of KSC with their personal illness narratives, fostering a swifter embracement of KSC.

#### Beneficial to reduce the economic burden

The fiscal strain engendered by chronic overtreatment is formidable, especially for those bereft of substantial financial cushions. Several patients identified this financial encumbrance as a catalyst for their gravitation towards KSC,a divorced rural woman explained,“I have no health insurance and no family support. I’m under a lot of pressure right now,a solitary rural woman, divorced and devoid of insurance, confided, “The weight of financial anxieties is overwhelming. If KSC promises economic respite, my acceptance is unequivocal.” (P17) A working professional, three years into dialysis treatment, candidly revealed, “A staggering two-thirds of my monthly remuneration is directed towards medical expenses. There’s an inherent irony in literally purchasing life. Given the choice, I’d rather allocate these funds towards experiences that truly elate and comfort me.“ This potent testimony reinforces the instrumental role of financial relief in bolstering the uptake of KSC.

#### Beneficial to improve the quality of life

Following extended therapeutic regimens, a cohort of patients exhibits hesitance towards the continuation of aggressive treatments, articulating a preference for refined prognostic management and proactive disease intervention that prioritizes elevating the quality of life. Patient P15 elucidated, “I harbor a deep-seated desire to eschew additional suffering as I approach life’s finale. In my twilight years, there’s a diminishing rationale for enduring unnecessary pain. If KSC can augment my life quality in this terminal phase, I am inclined towards its adoption.“ An academically inclined patient posited, “Excessive medical interventions in the closing chapters of life are often superfluous, serving merely to exacerbate discomfort.“(P16) With the global advocacy for euthanasia, particularly among terminal cancer patients, there’s a burgeoning recognition of the primacy of life quality over mere longevity. A patient with an extensive nine-year history of hemodialysis and a prior kidney replacement (P18) recounted the profound distress post-cardiac arrest and subsequent cardiopulmonary resuscitation. After introspective deliberations with her spouse about end-of-life directives, she evinced a profound resolve to face impending mortality and future health challenges with equanimity .

### Part II

### Prospective clinical merits of KSC

#### Augmenting quality of life

While Renal replacement therapies indeed prolong the lifespans of those with ESRD, they concurrently usher in a plethora of complications, sometimes debilitating to the extent that elderly patients find dialysis intolerable. KSC’s philosophy emphasizes life quality optimization, rigorous symptom management, and a persistent evaluation of life quality as the illness trajectory evolves. Consequently, Nurse 1 asserted the profound essence of KSC, proclaiming, “Mere extension of life, devoid of inherent quality, borders on the futile. It’s imperative we uphold patient autonomy and prioritize quality in the terminal stages.“

#### Alleviating economic strain

The financial implications of Renal replacement therapies are hefty, further compounded by China’s variable reimbursement policies contingent on regional nuances. As the disease trajectory waxes in severity, the concomitant medical expenditures escalate, imposing a formidable financial strain on both patients and their kin. KSC’s ethos underscores preemptive control and intervention, aiming to curtail complications, thereby tempering treatment-associated expenses. Nurse 4 recognized the pragmatic merits of KSC, noting, “The kidney, even in its advanced stages of compromise, remains amenable to judicious dietary regulations and pharmaceutical interventions, potentially deferring the need for dialysis and subsequently mitigating the financial implications.“ Echoing this sentiment, Nurse 7 articulated, “KSC facilitates elderly patients’ tranquil culmination of life’s journey within the familiar confines of home, without undue physical or financial strain, decisively alleviating the financial encumbrance on patients and their families.“

#### Effective use of medical resources

Given the considerable population of CKD patients in China juxtaposed with the heterogeneously distributed medical resources across its territories, there’s a pressing imperative to bolster symptom management. Strengthening and improving symptom management can stabilize disease progression while enhancing the health-related quality of life. Through the dual strategy of early diagnosis and proactive treatment, KSC unveils a platform of constructive support measures, aiming to judiciously distribute medical resources, ensuring patients use effectvie therapeutic interventions and care. Nurse 2 posits that KSC can diminish the drain on medical resources, concurrently attenuating the outflow from national medical insurance coffers. Concurrently, Nurse 5 articulated, “Although the KSC paradigm grapples with multifaceted challenges and debates, its potential in conserving medical resources remains undeniable.“

### Mltiple difficulties encountered

#### Treatment decision difficulty

The amplifying physiological and psychological encumbrances experienced during life’s twilight phases palpably erode the quality of life for ESRD patients. In this nuanced transition from affliction to life’s culmination, patients and their kin grapple with the vexing decision, persist with dialysis or transition to KSC. Steeped in traditional Chinese cultural tenets, direct dialogues about life’s finitude remain evasive for both patients and their families. Consequently, the choice to discontinue aggressive treatments becomes daunting. Doctor 2 observed, “Clinically, when broaching topics of mortality with patients or their kin, there’s a conspicuous predilection to circumvent these discussions.“ Similarly, Nurse 6 noted, “Rooted in our deep-seated familial values, numerous families find the decision to cease treatments profoundly challenging.“

#### The imperfection of the palliative system

The genesis of palliative care in mainland China is relatively nascent. The comprehension of this therapeutic approach, among healthcare professionals, patients, and their families, remains suboptimal. Particularly within nephrology, the palliative care framework exhibits gaps. Nurse 3 opined, “Given that hospitals primarily focus on disease amelioration and life preservation, KSC’s widespread adoption seems implausible. Contemporary Chinese medical institutions typically relegate palliative endeavors to specialized hospices or dedicated wards. Propagating this care approach in top-tier hospitals remains fraught with complexities.“ Echoing these sentiments, Doctor 1 emphasized, “Cessation of dialysis inevitably influences the patient’s life expectancy. Given the profound survivorship aspirations among patients and their families, KSC’s conservative approach could face resistance.“ Doctor 3 highlighted a crucial distinction, “The nuances of palliative care for Renal ailments differ markedly from oncological palliation. The evolution of palliative care for ESRD lags, with palpable deficits in awareness among both patients and their families, posing challenges for KSC’s broad-based implementation.”

## Discussion

The present investigation stands as a pioneering endeavor delving into the attitudes and perceptions of healthcare professionals, patients who have ESRD, in relation to KSC. Our findings elucidate a presently muted awareness amongst Chinese dialysis patients concerning KSC. Furthermore, healthcare professionals echo the sentiment that the progression of KSC for ESRD is riddled with challenges. Nevertheless, a subset of patients voiced a palpable need for KSC, extolling its potential to maintain quality of life during the terminal stages. Healthcare professionals, encompassing both physicians and nursing, corroborate the salience of KSC within the Chinese palliative care landscape.

The KSC paradigm, initially articulated in 2013 [26], underscores patient-centric endeavors aimed at mitigating disease-induced suffering, and maintaining (as much as possible) or improving quality of life for terminal patients, which is accomplished through prompt recognition, exhaustive evaluation, and a keen focus on patients’ physiological and psychological challenges. Historically, the momentum for KSC’s proliferation has been most pronounced in Canada, the United Kingdom, Australia, and New Zealand [27]. A plethora of scholarly contributions has rigorously dissected attitudes and perspectives pertaining to KSC’s application, meticulously analyzing the hurdles KSC encounters during its evolution, whilst spotlighting its intrinsic merits [28]. The intricate tapestry of Chinese traditional values has somewhat decelerated the momentum of palliative care, rendering KSC nascent in its journey. However, academics in both Hong Kong and Taiwan have delved fervently into Chinese cultural paradigms [15], in relation to mainland China lagging in this scholarly pursuit. Taiwan emerged as the inaugural Asian territory championing legislation endorsing “natural death”, which is paralleled by the Hong Kong administration’s endorsement of palliative care. Empirical studies substantiate that advancing palliative care can enhance patients’ understanding of their clinical trajectories, resulting in an uplift in their quality of life [7]. Concurrently, it portends a reduction in superfluous medical interventions and unwarranted resource utilization, thereby furnishing valuable insights for the maturation of palliative care in mainland China [29].

Interviewed patients in this study, albeit possessing limited KSC knowledge, displayed anoptimistic and receptive disposition. The ESRD patients who participated in this study manifested a pronounced appetite for palliative care. However, the extant medical provisions and societal support in China remain wanting [30]. There exists a discernible reticence among Chinese patients towards proactive participation in medical decision-making [31]. Furthermore, throughout their clinical journeys, patients and their kin tend to deify healthcare professionals, conspicuously lacking in prognostic acumen and collaborative decision-making endeavors with physicians [32].Therefore, the clinical application and development of KSC in China is still facing more difficulties.

KSC represents a salient therapeutic approach that, through early identification, seeks to mitigate and ameliorate the profound challenges faced by patients confronted with life-threatening afflictions [33]. Yet, within the cultural tapestry of traditional Chinese society, preemptive dialogues concerning prognosis and palliative care often meet with resistance and reticence [34]. Furthermore, the prevailing medical decision-making ethos leans more towards a paternalistic paradigm rather than one of collaborative deliberation [35], thereby attenuating patient autonomy in clinical choices. Consequently, KSC’s reception among Chinese patients remains tepid.

Healthcare professionals consulted in this research expounded their endorsement of KSC’s value in the domain of kidney diseases. However, they also voiced concerns about the inherent quandaries impeding KSC’s evolution within mainland China. Charting the trajectory of KSC in China necessitates a nuanced exploration, intertwining it with the nation’s rich cultural heritage. Foremost, there is an imperative to amplify public educational endeavors, aimed at fostering enhanced understanding and advocacy for palliative care. This entails the inauguration of dedicated healthcare professionals specializing in Renal disorders and crafting hierarchical organizational structures spanning from tertiary healthcare facilities to grassroots communities [36]. Leveraging these professional cohorts to champion public initiatives can stimulate patient and familial involvement in KSC-centric dialogues [37]. Furthermore, to accentuate the directive influence of regulatory frameworks, governmental agencies should promote policies bolstering palliative care, thereby refining the caliber of elderly care services [38]. This would involve delineating distinct responsibilities amongst state entities, societal structures, and familial units, furnishing policy-driven impetus for the specialized palliation of Renal conditions. In tandem, there’s a pressing need to weave KSC into the fabric of our indigenous culture. This involves carefully gauging the aspirations of Chinese Renal patients in relation to KSC, pinpointing the most efficacious method for KSC’s clinical application, thereby fulfilling the expectations of patients and their kin most holistically [39]. Finally, international cooperation should be strengthened to further determine the development path of KSC, optimize the admission criteria and implementation content of KSC, and reach international consensus on the application of KSC, so as to promote the consistency of clinical practice, research and policy.

### Limitations

While our study is not without limitations, it is essential to acknowledge the inherent constraints within our research. First and foremost, we must consider the participants who were included in our study as well as those who were excluded. Notably, individuals with ESRD who were not undergoing dialysis were absent from our study sample. This omission raises crucial questions about the scope of our research and the reasons behind excluding this particular group.

Furthermore, even though our study collected data on patients’ marital status, it did not delve into their living arrangements. We did not gather information about whether they were living with others, residing in nursing homes, or living independently. This distinction is significant because marital status alone may not provide a comprehensive explanation for the dynamics we sought to understand.

To offer a more comprehensive perspective, we must engage in critical self-reflection regarding our work. This process should involve identifying and scrutinizing other potential limitations that may have influenced our research findings. Additionally, it’s worth noting that our research was geographically limited to Beijing and Zhengzhou, resulting in gaps in insights from other urban areas and medical institutions. The small sample size of the study may somewhat limit its representativeness in the field of medical research. Therefore, in future endeavors, there is an urgent need to increase the sample size and complement the current research findings with more extensive research efforts.

## Conclusion

Our research results emphasize the significant acceptability of KSC.The data gleaned underscores a noticeable acceptability of KSC. Collectively, interviewees acknowledged KSC’s instrumental role in ameliorating patients’ symptomatology and enhancing their overall quality of life. Concurrently, both patients and healthcare professionals discern a strong potential in KSC’s integration into clinical paradigms, championing patient-centric care, and orchestrating therapeutic interventions that resonate with patients’ ethical compass, preferences, and long-term objectives via collaborative decision-making and strategies for anticipatory care.

Moreover, the study delineates existent impediments to the efficacious rollout of KSC within the Chinese milieu, such as limited patient cognizance, a nascent medical infrastructure tailored for KSC, and tepid policy endorsements. The journey of embedding KSC within China’s healthcare system is still in its infancy. There exists an exigent mandate to amplify KSC’s outreach within our nation, refining the awareness of Renal patients and healthcare providers, and assiduously enhancing the scaffolding that underpins it.

## Data Availability

The data that support the findings of this study are available from the corresponding author, Luo, upon reasonable request.
